# Infrared Camera-Based Non-contact Measurement of Brain Activity From Pupillary Rhythms

**DOI:** 10.3389/fphys.2018.01400

**Published:** 2018-10-10

**Authors:** Sangin Park, Mincheol Whang

**Affiliations:** ^1^Industry-Academy Cooperation Team, Sangmyung University, Seoul, South Korea; ^2^Department of Intelligent Engineering Informatics for Human, Sangmyung University, Seoul, South Korea

**Keywords:** vital sign monitoring, brain activity, pupil size variation, harmonic frequency, non-contact measurement

## Abstract

Pupillary responses are associated with affective processing, cognitive function, perception, memory, attention, and other brain activities involving neural pathways. The present study aimed to develop a noncontact system to measure brain activity based on pupillary rhythms using an infra-red web camera. Electroencephalogram (EEG) signals and pupil imaging of 70 undergraduate volunteers (35 female, 35 male) were measured in response to sound stimuli designed to evoke arousal, relaxation, happiness, sadness, or neutral responses. This study successfully developed a real-time system that could detect an EEG spectral index (relative power: low beta in FP1; mid beta in FP1; SMR in FP1; beta in F3; high beta in F8; gamma P4; mu in C4) from pupillary rhythms using the synchronization phenomenon in harmonic frequency (1/100 f) between the pupil and brain oscillations. This method was effective in measuring and evaluating brain activity using a simple, low-cost, noncontact system, and may be an alternative to previous methods used to evaluate brain activity.

## Introduction

Human vital signs have been used to assess human behavior in various fields, including ubiquitous health care, emotional information and communication technology, emotional recognition and engineering, human computer interaction, and security (Park et al., 2018). However, sensor-based measurement methods have a significant disadvantage because attachment of sensor devices to the human body is required. Subjects may experience pressure and discomfort when sensors are attached to their body or skin, and these limitations restrict their direct application in industrial and medical fields. Moreover, these sensors are expensive and bulky.

Vital sign monitoring technology has recently been incorporated into wearable wireless devices and have advanced the development of portable measuring equipment. These wearable devices should be manufactured in the form of accessory items, such as watches, bracelets, or glasses that measure heart rate (HR), respiration, skin temperature, and galvanic skin responses. These devices have the advantages of convenience and less burden on users for sensor attachment compared with previous wire-based sensors. However, these sensors are expensive and are susceptible to noise caused by movement. Thus, noncontact, low-cost measurement methods to detect physiological signals unaffected by movement for various applications in industrial and medical fields are required (Boric-Lubeke and Lubecke, 2002; Suzuki et al., 2009; Poh et al., 2011; Scully et al., 2012; Balakrishnan et al., 2013; Tarassenko et al., 2014; Park et al., 2018).

Considering these limitations and requirements, noncontact measurement methods have recently been proposed and developed by many researchers. Previous studies have measured cardiac activity and respiration using radar, thermal imaging, web-camera (web-cam), and infrared (IR) camera. Microwave and Doppler radars measure HR and respiration rate by sensing movement of the chest during inspiration and expiration (Droitcour et al., 2001; Lohman et al., 2002; Uenoyama et al., 2006; Fletcher and Han, 2009; Li et al., 2009; Suzuki et al., 2009; Gu et al., 2010; Kao et al., 2013; Lim et al., 2015). Thermal imaging uses temperature variations in the wrist, face (nasal), and neck (carotid artery and jugular vein) caused by blood flow moving from the heart to the brain to monitor HR (Chekmenev et al., 2005, 2009; Sun et al., 2006; Garbey et al., 2007; Gault et al., 2010) and respiration rate (Chekmenev et al., 2005; Murthy and Pavlidis, 2005; Murthy et al., 2009; Fei and Pavlidis, 2010; Abbas et al., 2011). Web-cams detect color variations in the face, and micro-movements in the head and chest caused by blood flow and expiration-inspiration, and extract information related to cardiac activity and respiration from these factors (Poh et al., 2011; Balakrishnan et al., 2013; Holton et al., 2013; Janssen et al., 2015). Pupillary rhythm measured using IR cameras is a reflection of autonomic nervous system (ANS) activity, and related to cardiac activity and blood pressure (Loewenfeld and Lowenstein, 1993; Steinhauer et al., 2000; Verney et al., 2001; Siegle et al., 2004; Park et al., 2018). Many previous studies have reported that pupillary rhythm is synchronized with HR variability (Calcagnini et al., 1997; Hung and Zhang, 2006; Onorati et al., 2013; Parnandi and Gutierrez-Osuna, 2013; Park et al., 2018), blood pressure variation (Hung and Zhang, 2006; Bär et al., 2009), and respiration rhythm (Calcagnini et al., 1997). Previous studies have developed methods for non-contact measurement of physiological signals using each of the above modalities. These technologies can accurately measure multiple physiological cardiac-, respiratory-, and blood pressure-related parameters through a simple, low cost, non-contact measurement system, which have applications in various fields of industry and medicine. However, previous studies have not considered brain activity, such as electroencephalogram (EEG) spectra, for noncontact measurement. The EEG spectrum is also a human physiological response that is as highly used and extensively studied as other physiological signals. However, measuring EEG signals is an inconvenient, complex and, at times, onerous process, and requires the user to wear specialized sensors. Therefore, noncontact technology in this context requires significant research effort.

The behavior of the pupils reflects physiological and neurological mechanisms in humans, and responses in the pupil can be measured using cameras (i.e., noncontact). Additionally, the pupillary response is strongly related to central nervous system activity (i.e., functional brain processing) and based on afferent and efferent neural-pathways (Kahneman and Beatty, 1966; Beatty and Lucero-Wagoner, 2000; Fotiou et al., 2000; Partala and Surakka, 2003; Kojima et al., 2004; Kozicz et al., 2011; Prehn et al., 2011; Júnior et al., 2015). In the afferent pathway (i.e., CN II) from retinal receptors, when the pupil is stimulated by light input, the retina (rod and cone cells) synapse with their respective bipolar cells (the primary neuron), and this signal transmits to ganglion cells (the secondary neuron). And then the ganglion cell axons project an electrical signal through the optic nerve to the ipsilateral pretectal nucleus of the mid-brain. In the efferent pathway (i.e., CN III) from the mid brain, the pretectal nucleus is connected to the Edinger-Westphal nucleus (Loewenfeld and Lowenstein, 1993; Steinhauer et al., 2000; Verney et al., 2001; Siegle et al., 2004; Prehn et al., 2011). For example, the pupillary light reflex is known to be a clinical indicator for assessing mid-brain function such as the connectivity between the Edinger-Westphal nucleus and sphincter muscle in the pupil (Fotiou et al., 2000; Kojima et al., 2004; Kozicz et al., 2011; Júnior et al., 2015), and it is related to the stimulation from the pupillary smooth muscles of the sphincter muscle to cause pupil contraction (Maggs et al., 2012). In addition, previous studies have reported that pupillary rhythm (pupil diameter) is related to affective processing (Surakka et al., 1998; Partala and Surakka, 2003), cognitive function (Hess and Polt, 1964; Kahneman and Beatty, 1966; Ahern and Beatty, 1979; Just and Carpenter, 1993; Just et al., 1996; Klingner et al., 2008), perception (Hakerem and Sutton, 1966; Norman and Bobrow, 1975), memory (Beatty and Kahneman, 1966; Kahneman and Beatty, 1966; Just and Carpenter, 1993; Just et al., 1996), attention (Hink et al., 1977; Beatty, 1988), and brain activity (Just and Carpenter, 1993; Just et al., 1996).

The EEG spectrum is known to be an indicator in assessing brain processing in the above-mentioned areas (van Beijsterveldt and Boomsma, 1994; Dietrich and Kanso, 2010), and these brain functions have been shown to be reflected in pupillary rhythms in previous studies. We hypothesized that pupillary rhythm is synchronized and connected with brain activity. The aim of this study, therefore, was to measure brain activity (reflected by EEG spectra) by examining pupillary rhythm using a vision-based, noncontact measurement system. For this purpose, this study analyzed synchronization in each frequency range (e.g., delta, theta, alpha, and beta) between the pupil and EEG rhythms based on harmonic frequency. The technique represents a new and more advanced non-contact method of measuring brain activity that uses an IR web-cam to image the pupils.

## Methods

### Participants

Seventy undergraduate volunteers (35 male, 35 female), ranging in age from 22 to 28 years (mean age, 23.46 ± 0.82 years), participated in the present study. All participants were right-handed, and had normal or corrected-to-normal vision (i.e., participant's vision >0.8), and no family or medical history of disease affecting their visual functionality or central nervous system. In addition, participants were required to abstain from alcohol, smoking, and caffeine for at least 12 h before the experiment, and to sleep according to their normal schedule. The participants were notified of the above restrictions and requirements, and provided informed written consent before being tested. This study was carried out in accordance with the recommendations of Sangmyung University Institutional Bioethics Review Board (SMUIBRB), Seoul, South Korea (No. BE2015-7-1). The protocol was approved by the SMUIBRB.

### Stimuli, experimental protocol, and data acquisition

**Figure 1:**
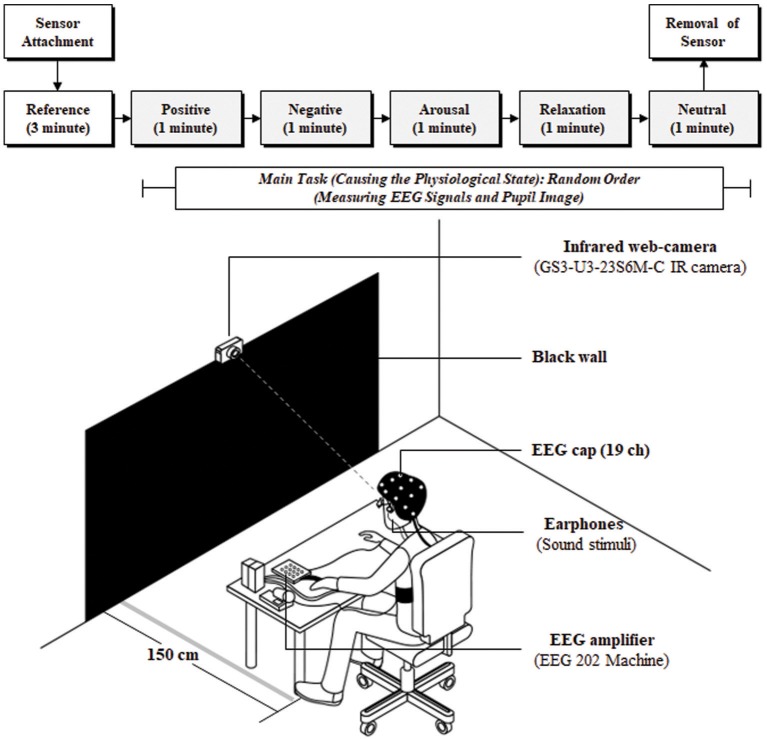
Experimental protocol and configuration. EEG, electroencephalogram; IR, infra-red.

The experiment involved reference and main-task stimuli. The reference was presented for 3 min before the main task to stabilize physiological state without stimulus. In the main task, sound stimuli invoking arousal, relaxation, positive, negative, and neutral responses were presented in random order to participants for 5 min, with each stimulus lasting 1 min. The stimulus was selected to be sound instead of video to minimize the effect of illumination on the pupil. The participants gazed at a black wall, placed at a distance of 1.5 m, while sitting in a comfortable chair listening to the sound stimuli. Pupil imaging and EEG signals were measured simultaneously during the experiment, as shown in Figure 1. The black wall and sound stimuli were selected to minimize the influence of illumination changes on pupil size variation (PSV) because pupil size is strongly affected by these variations.

This study considered changes in physiological state in the experimental design because the EEG spectrum is well known to be strongly influenced by physiological conditions. Stimuli comprised five components including arousal, relaxation, and positive, negative, and neutral based on the cir-complex model proposed by Russell (1979) to provoke physiological variation in the participants. Stimuli causing physiological state variation were similar to those applied in previous studies with the following specifications: the stimuli sources were recorded for each emotion; the stimuli were categorized into five groups (arousal, relaxation, positive, negative, and neutral); a chi-squared test to assess goodness-of-fit, based on subjective ratings from 150 subjects; principal component analysis was based on varimax (orthogonal) rotation from 150 subjects (seven-point scale); and representative stimuli for each emotion were selected (sound files in Supplementary Material; Park et al., 2018). The EEG signals were recorded at a 500 Hz sampling rate from 19 channels (FP1, FP2, F3, Fz, F4, F7, F8, C3, Cz, C4, T7 [T3], T8 [T4], P7 [T5], P8 [T6], P3, Pz, P4, O1, and O2 regions) based on the international 10–20 system (ground: FAz; reference: average between electrodes on the two ears; and DC level: 0–150 Hz) using an EEG device (202, Mitsar Inc., Russia). Pupil images were recorded at 125 fps with a resolution of 960 × 400 using an IR camera (GS3-U3-23S6M-C, Point Gray Research Inc., Canada).

### Assessment of brain activity from pupillary response

#### Detecting brain activity from EEG signals (ground truth)

**Figure 2:**
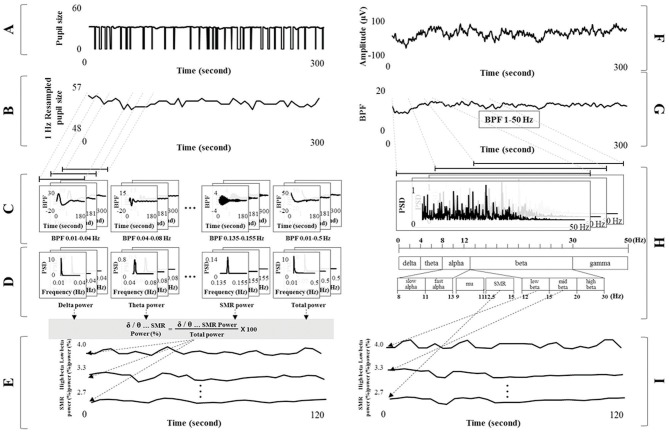
Signal processing for detecting the electroencephalogram (EEG) spectral index from pupillary response and electrocardiograph signals. **(A)** Pupil diameter at 125 fps. **(B)** Resampled pupil diameter at 1 Hz based on sliding moving average (window size: 125 fps and shift: 125 fps). **(C)** Band pass filtered signals of each frequency band from the delta to SMR band with harmonic frequency of 1/100f. **(D)** Spectral power for each band from pupillary rhythm using fast Fourier transform analysis. **(E)** Relative powers calculated using the ratio between total and each band power. **(F)** Raw EEG signals. **(G)** Band pass filtered signals of 1–50 Hz. **(H)** EEG spectrum analysis and extraction of each band power (from delta to SMR). **(I)** Relative power of each band from EEG signals (ground truth).

EEG signals were processed using a band pass filter (BPF, Butterworth type of order 6) of 1–50 Hz, and the EEG spectrum was analyzed using a fast Fourier transform (FFT) method. The EEG spectrum was divided according to frequency band into the following ranges: delta 1–4 Hz; theta 4–8 Hz; alpha 8–13 Hz; beta 13–30 Hz; gamma 30–50 Hz; slow alpha 8–11 Hz; fast alpha 11–13 Hz; low beta 12–15 Hz; mid beta 15–20 Hz, high beta 20–30 Hz, mu 9–11 Hz; the SMR 12.5–15.5 Hz; and total power 1–50 Hz (Ramaekers et al., 1992; Deuschl and Eisen, 1999; Berta et al., 2013; Shin et al., 2014; Novais and Konomi, 2016). Each band power was extracted. The relative powers from each of the frequency bands were then calculated using the ratio between the total band power and each band power. This procedure was executed using the sliding moving average with a window size of 30 s and a shift of 1 s, as shown in Figure 2.

#### Detecting brain activity from pupil signals

The pupil area was calculated using the method described in previous studies (Daugman, 2004; Lee et al., 2009; Park et al., 2018). Gray-scale images captured from an IR camera were processed using binarization with a threshold value, as shown in Equation (1). This equation for binarization was extracted from a linear regression model between mean and maximum brightness value from the entire image in a previous study (Park et al., 2018). The pupil position from the binarized image was then calculated using a circular edge detection algorithm (Daugman, 2004; Lee et al., 2009), as shown in Equation (2).

(1) $$\begin{array}{lll} Threshold\;for\;binarization & = & \left(-0.418\times B_{mean}\right)\\ \;+\left(1.051\times B_{\text{max}}\right)+7.973 \end{array}$$

(2) $$\begin{array}{r} Max_{\left(r,\;x_{0},\;y_{0}\right)}|G\sigma \left(r\right)*\frac{\partial }{\partial r}\oint_{r,\;x_{0},\;y_{0}}\frac{I\left(x,y\right)}{2\pi r}\text{d}s| \end{array}$$

**Figure 3:**
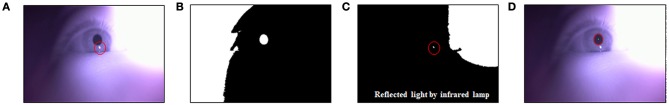
Protocol for measuring the pupil area. **(A)** A gray-scale image from the infrared web-cam. **(B)** Binarization image based on auto threshold. **(C)** Detecting the reflected light caused by the infrared lamp. **(D)** Calculating pupil position using a circular edge detection algorithm.

In Equation (1), *B*_mean_ and *B*_max_ denote the mean and maximum brightness value from the entire image in gray scale, respectively. In Equation (2), *I*(*x, y*) indicates a gray level at the (*x, y*) position, and (*x*_0_, *y*_0_) and *r* represent the center position and radius of the pupil, respectively. *Gσ*(*r*) represents a smoothing function to detect a circular edge blurred at a scale set by σ (Gaussian type). An accurate pupil position was identified by the reflected light caused by the IR lamp when multiple pupil positions were selected, as shown in Figure 3.

The process of assessing brain activity (i.e., EEG spectral power for each band) was as follows. Pupil diameter was processed using the sliding moving average (i.e., window size of 1 s and shift of 1 s) from 125 to 1 fps (1 Hz resampling). For example, a pupil diameter of 125 fps was calculated by mean value (1 fps). The moving average procedure was applied in the proposed method because pupil area was not detected during eye closure. Pupil diameter can be acquired using this method if the time the eyes are closed is < 1 s. However, non-tracked pupil diameters caused by the eye closing >1 s were not involved in the moving average procedure. The resampled pupil diameter was processed using the BPF (Butterworth type of order 6) into separate frequency bands of delta (0.01–0.04 Hz), theta (0.04–0.08 Hz), alpha (0.08–0.13 Hz), beta (0.13–0.30 Hz), gamma (0.30–0.50 Hz), slow alpha (0.08–0.11 Hz), fast alpha (0.11–0.13 Hz), low beta (0.12–0.15 Hz), mid beta (0.15–0.20 Hz), high beta (0.20–0.30 Hz), mu (0.09–0.11 Hz), SMR (0.125–0.155 Hz), and total band (0.01–0.50 Hz). These frequency bands in pupillary rhythm were applied to the harmonic frequency band with a 1/100 ratio from the EEG spectrum band, assuming that the pupillary and brain rhythms were synchronized. Filtered signals for each frequency band (from delta to total band) were proceeded using FFT analysis to change into the spectrum from time serial data. Each band power was defined by the summation of all frequency power value from each spectrum data. The relative powers of each frequency band, from delta to SMR, were calculated using the ratio between the total and each band power, as shown in Equation (3). This procedure was performed using the sliding moving average with a window size of 30 s and a shift of 1 s, as shown in Figure 2.

(3) $$\begin{array}{r} Relative\;power\;\left(\%\right)=\;\frac{Each\;band\;power}{Total\;band\;power}\times 100 \end{array}$$

### Statistical analysis

This study measured the EEG spectral index from pupillary rhythm, represented by delta, theta, alpha, beta, gamma, slow alpha, fast alpha, low beta, mid beta, high beta, mu, and SMR relative power for the 19 channel brain regions, based on the synchronization between the brain and pupillary rhythms. Relative powers for EEG spectral index extracted from pupillary rhythm were compared with the EEG spectral index from brain activity (ground truth) based on the correlation analysis (Pearson correlation coefficient, *r*), mean error value (*ME*), and Bland-Altman plot (difference plot). The correlation coefficient ranging in value from −1 to 1 was defined by the linear relationship between two or more variables. A correlation coefficient approaching a value of −1 indicates a strong negative correlation, and that approaching a value of 1 indicates a strong positive correlation (Stigler, 1989). Mean error was defined as the difference in value of the relative powers of each band between pupil and brain. A Bland-Altman plot (mean-difference plot) is a data plotting method for assessing agreement between two methods. Bland and Altman make the point that any two methods that are designed to measure the same parameter (or property) should have good correlation when a set of samples are chosen such that the property to be determined varies considerably. This plot consists of the x and y axes, and correspond to the mean and difference values between two measures, respectively. If most of the measured values from two variables are within $\bar{d}\pm 2s$ (more precisely, between $\bar{d}+1.96s$ and $\bar{d}-1.96\;s$: a 95% confidence level) in a Bland-Altman plot, the two measures have agreement, where $\bar{d}$ and *s* are mean difference and standard deviation, respectively (Bland and Altman, 1986). All statistical analyses were performed using SPSS Statistics version 19.0 (IBM Corporation. Armonk, NY, USA).

## Results

**Figure 4:**
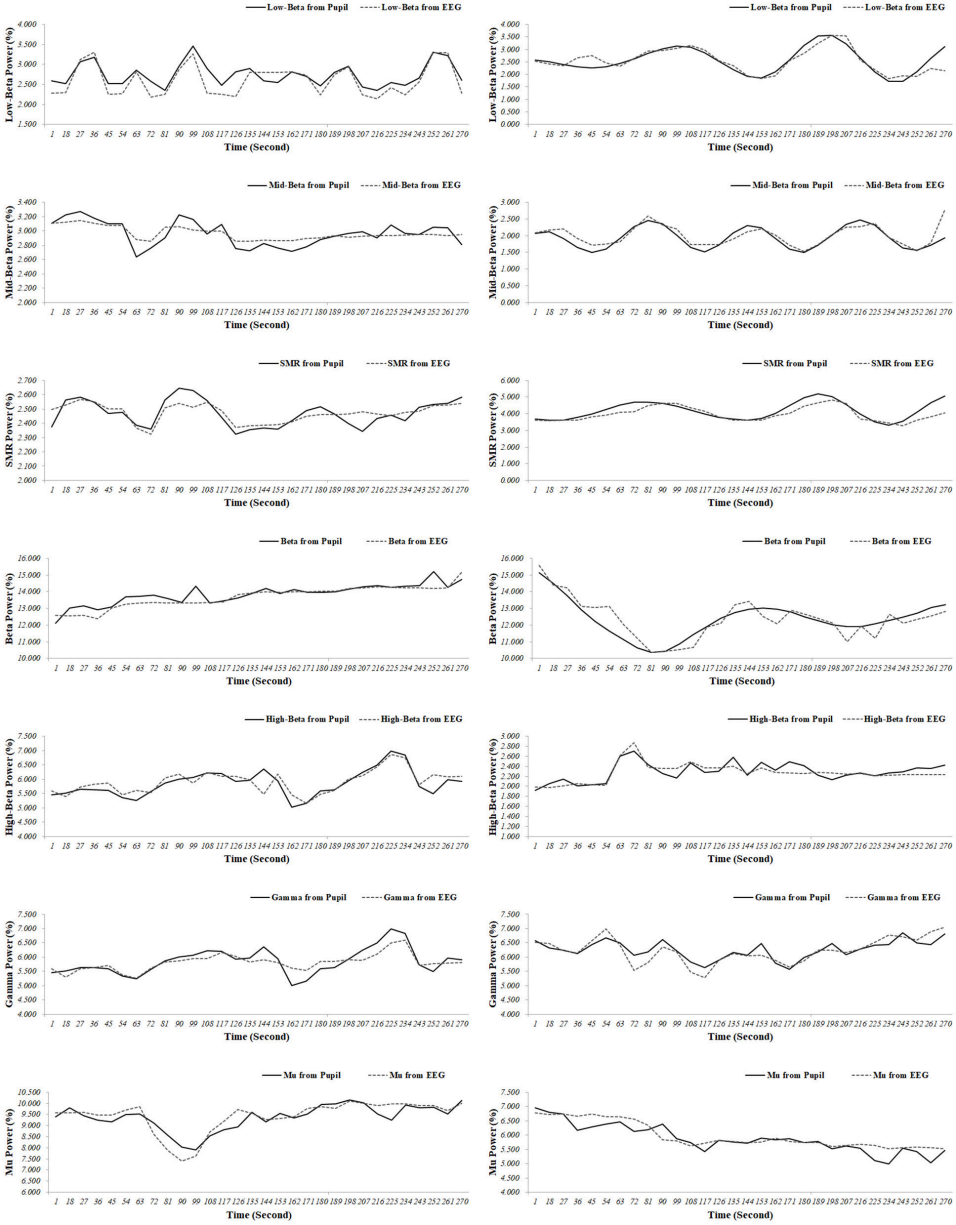
Representative examples (participant 6 [left panel]; participant 42 [right panel]) of the electroencephalogram (EEG) spectral index from the pupil and EEG signals (low beta power in the FP1 region; mid beta power in the FP1 region; SMR power in the FP1 region; beta power in the F3 region; high beta power in the F8 region; gamma power in the P4 region; mu power in the C4 region).

Representative examples of an extracted EEG spectral index from the pupillary response, and the EEG signals for the test participants (participants 6 and 42) are shown in Figure 4. When comparing the results for the ground truth, the EEG spectral index from the pupillary response demonstrated a strong positive correlation with EEG signals for seven parameters (participants 6 and 42), in which *r* = 0.863 (*p* < 0.001) and 0.862 (*p* < 0.001) for low beta power in the FP1 region; *r* = 0.853 (*p* < 0.001) and 0.803 (*p* < 0.001) for mid beta power in the FP1 region; *r* = 0.800 (*p* < 0.001) and 0.840 (*p* < 0.001) for SMR power in the FP1 region; *r* = 0.857 (*p* < 0.001) and 0.882 (*p* < 0.001) for beta power in the F3 region; *r* = 0.826 (*p* < 0.001) and 0.838 (*p* < 0.001) for high beta power in the F8 region; *r* = 0.882 (*p* < 0.001) and 0.866 (*p* < 0.001) for gamma power in the P4 region; *r* = .882 (*p* < 0.001) and 0.868 (*p* < 0.001) for mu power in the C4 region. This result processed using the sliding moving average with a window size of 30 s and a shift of 1 s measurement over a 300 s interval.

**Figure 5:**
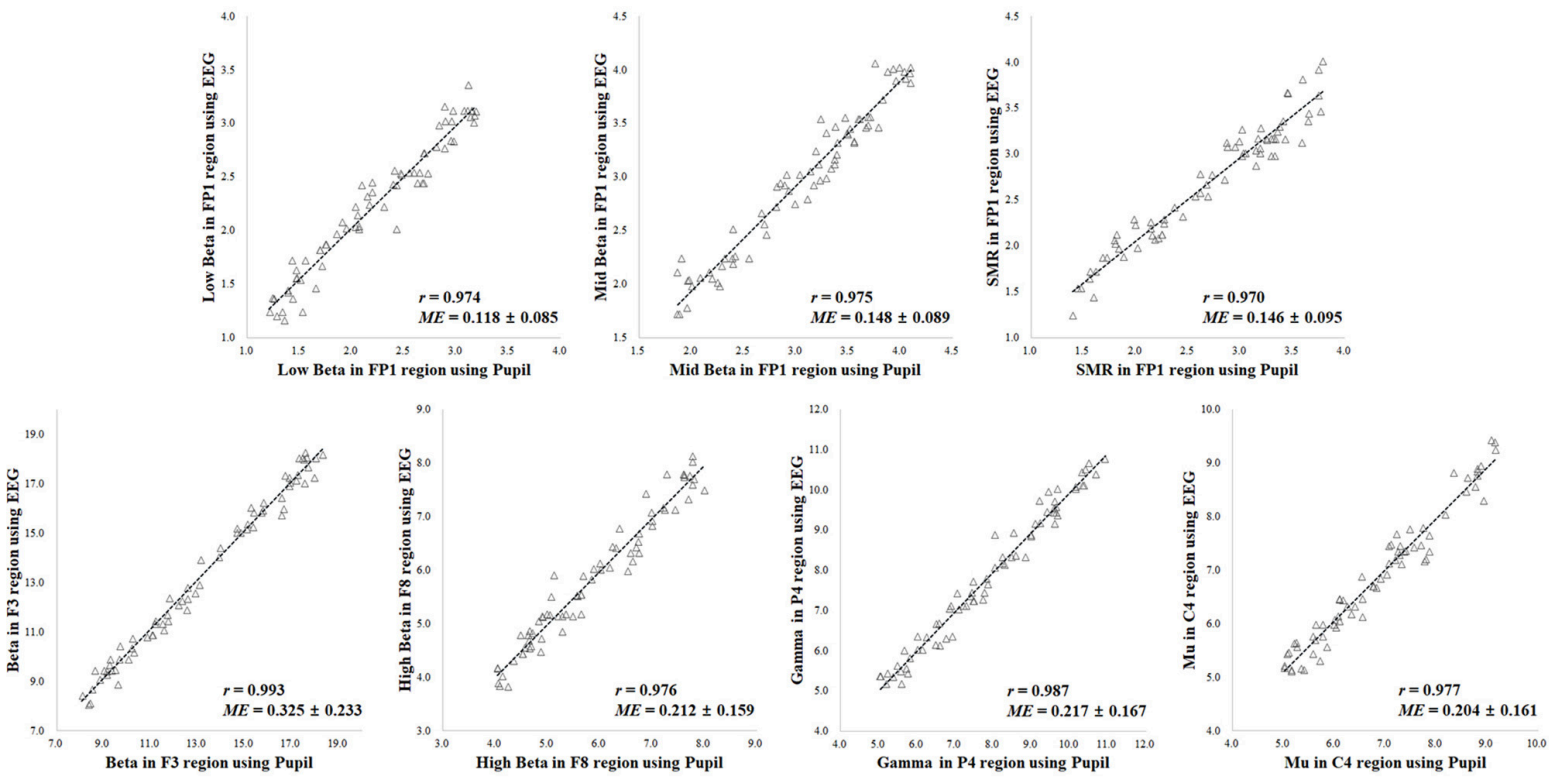
Results of correlation analysis in reference state for coefficient (*r*) and mean error (*ME*) of electroencephalogram (EEG) spectral index between the pupil and EEG signals (*p* < 0.001 [*n* = 70]).

A comparison of the results for the 70 participants in reference state (i.e., neutral) is shown in Figure 5. When comparing the results of the ground truth, the EEG spectral index from the pupillary response demonstrated a strong positive correlation (0.970–0.993) and low mean error (0.118–0.325) with EEG signals for seven parameters, in which *r* = 0.974 (*p* < 0.001) and *ME* = 0.118 ± 0.085 for low beta power in the FP1 region; *r* = 0.975 (*p* < 0.001) and *ME* = 0.148 ± 0.089 for mid beta power in the FP1 region; *r* = 0.970 (*p* < 0.001) and *ME* = 0.146 ± 0.095 for SMR power in the FP1 region; *r* = 0.993 (*p* < 0.001) and *ME* = 0.325 ± 0.233 for beta power in the F8 region; *r* = 0.976 (*p* < 0.001) and *ME* = 0.212 ± 0.159 for high beta power in the F8 region; *r* = 0.987 (*p* < 0.001) and *ME* = 0.217 ± 0.167 for gamma power in the P4 region; *r* = 0.977 (*p* < 0.001) and *ME* = 0.204 ± 0.161 for mu power in the C4 region.

**Figure 6:**
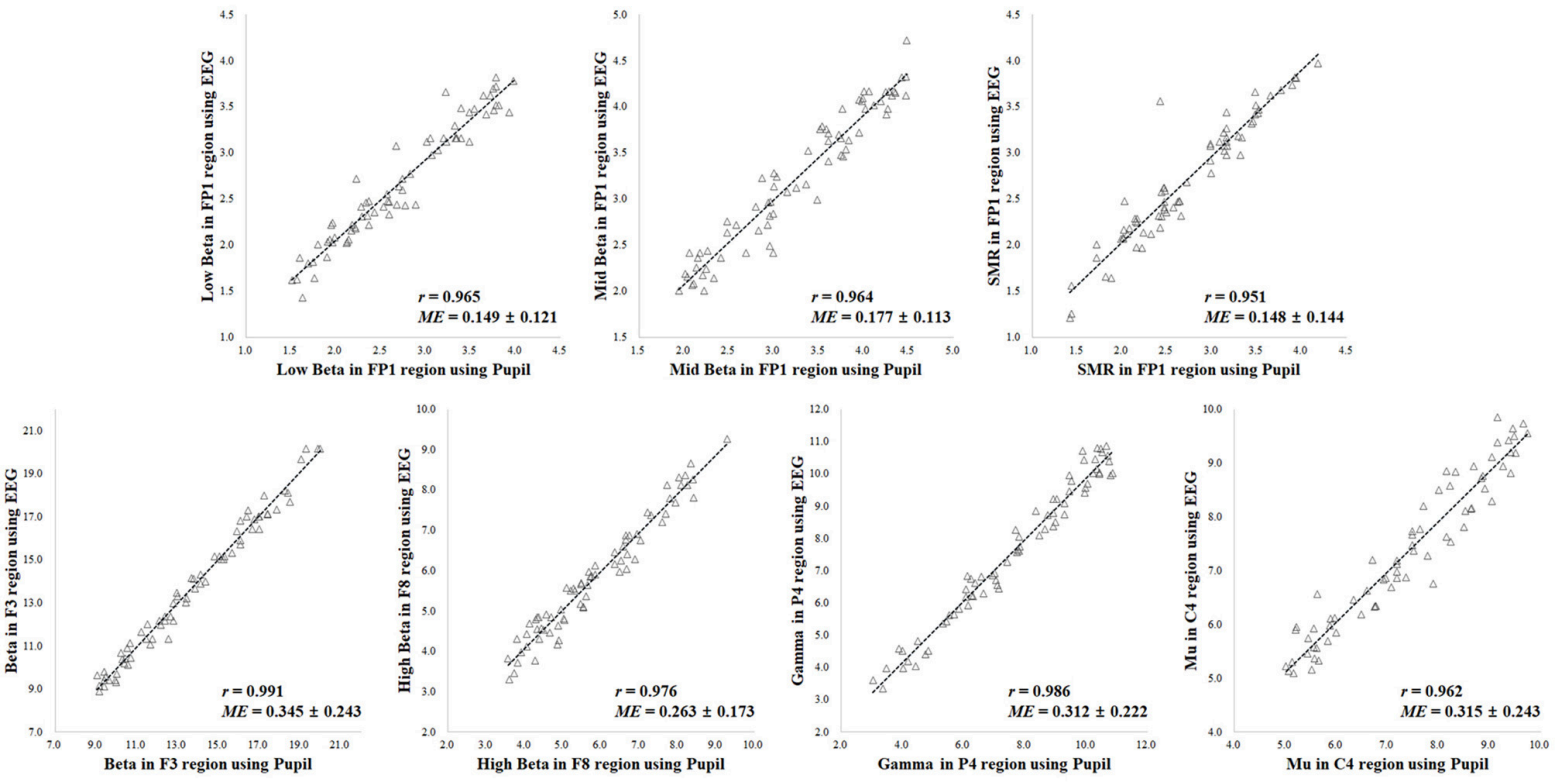
Results of correlation analysis in variation of physiological state for coefficient (*r*) and mean error (*ME*) of electroencephalogram (EEG) spectral index between the pupil and EEG signals (*p* < 0.001 [*n* = 70]).

A comparison of the results for the 70 participants in variation of physiological state (i.e., positive, negative, arousal, and relaxation) is shown in Figure 6. When comparing the results of the ground truth, the EEG spectral index from the pupillary response demonstrated a strong positive correlation (0.951–0.991) and low mean error (0.148–0.345) with EEG signals for seven parameters, in which *r* = 0.965 (*p* < 0.001) and *ME* = 0.149 ± 0.121 for low beta power in the FP1 region; *r* = 0.964 (*p* < 0.001) and *ME* = 0.177 ± 0.113 for mid beta power in the FP1 region; *r* = 0.951 (*p* < 0.001) and *ME* = 0.148 ± 0.144 for SMR power in the FP1 region; *r* = 0.991 (*p* < 0.001) and *ME* = 0.345 ± 0.243 for beta power in the F8 region; *r* = 0.976 (*p* < 0.001) and *ME* = 0.263 ± 0.173 for high beta power in the F8 region; *r* = 0.986 (*p* < 0.001) and *ME* = 0.312 ± 0.222 for gamma power in the P4 region; *r* = 0.962 (*p* < 0.001) and *ME* = 0.315 ± 0.243 for mu power in the C4 region. Result for the reference condition of correlation coefficient and mean error is slightly higher and lower than the condition of physiological variation. However, these results in both conditions of reference and physiological variation represented the brain region showing the highest correlation and lowest mean error for each frequency band with ground truth. The aforementioned results revealed the highest correlation and the lowest error among analysis results for all EEG frequency bands in each brain region, and the overall results are shown in Table 1. Results for the other frequency bands including delta, theta, alpha, slow and fast alpha were not statistically significant.

**Table 1:** Results of Pearson correlation coefficient and mean error in reference (physiological variation) for electroencephalogram (EEG) spectral index between the pupil and EEG signals (*N* = 70, *p* < 0.05).

	**D**	**T**	**A**	**B**	**G**	**SA**	**FA**	**LB**	**MB**	**HB**	**Mu**	**SMR**
**PEARSON CORRELATION COEFFICIENT (r)**												
FP1	–	–	–	0.467 (0.394)	–	–	–	**0.974 (0.965)**	**0.975 (0.964)**	0.432 (0.417)	–	**0.970 (0.951)**
FP2	–	–	–	0.412 (0.386)	–	–	–	0.882 (0.872)	0.907 (0.869)	0.482 (0.487)	0.272 (0.244)	0.847 (0.845)
F3	–	–	–	0.589 (0.586)	–	–	–	0.424 (0.416)	0.462 (0.452)	0.477 (0.482)	0.263 (0.257)	0.407 (0.371)
Fz	–	–	–	0.516 (0.533)	–	–	–	0.394 (0.372)	0.454 (0.461)	0.443 (0.413)	–	0.377 (0.364)
F4	–	–	–	0.626 (0.614)	–	–	–	0.406 (0.391)	0.432 (0.398)	0.520 (0.532)	0.312 (0.277)	0.342 (0.359)
F7	–	–	–	0.846 (0.882)	–	–	–	0.714 (0.721)	0.726 (0.717)	0.876 (0.882)	–	0.694 (0.711)
F8	–	–	–	**0.993 (0.991)**	–	–	–	0.698 (0.683)	0.715 (0.704)	**0.976 (0.976)**	–	0.672 (0.689)
C3	–	–	–	–	–	–	–	–	–	–	0.881 (0.854)	–
Cz	–	–	–	–	–	–	–	–	–	–	0.812 (0.804)	–
C4	–	–	–	–	–	–	–	–	–	–	**0.977 (0.962)**	–
T7(T3)	–	–	–	–	–	–	–	–	–	–	–	–
T8(T4)	–	–	–	–	–	–	–	–	–	–	–	–
P7(T5)	–	–	–	–	–	–	–	–	–	–	–	–
P8(T6)	–	–	–	–	–	–	–	–	–	–	–	–
P3	–	–	–	–	0.906 (0.882)	–	–	–	–	0.311 (0.326)	–	–
Pz	–	–	–	–	0.778 (0.741)	–	–	–	–	0.244 (0.257)	–	–
P4	–	–	–	–	**0.987 (0.986)**	–	–	–	–	0.276 (0.232)	–	–
O1	–	–	–	–	–	–	–	–	–	–	–	0.311 (0.256)
O2	–	–	–	–	–	–	–	–	–	–	–	0.293 (0.311)
**MEAN ERROR (ME)**												
FP1	–	–	–	0.807 (0.824)	–	–	–	**0.118 (0.149)**	**0.148 (0.177)**	0.663 (0.627)	–	**0.146 (0.148)**
FP2	–	–	–	–	–	–	–	0.167 (0.169)	0.176 (0.189)	0.692 (0.704)	–	0.202 (0.247)
F3	–	–	–	0.757 (0.774)	–	–	–	0.724 (0.776)	0.662 (0.643)	0.414 (0.473)	0.846 (0.927)	0.519 (0.494)
Fz	–	–	–	0.712 (0.742)	–	–	–	0.695 (0.702)	0.651 (0.665)	0.449 (0.452)	–	0.632 (0.667)
F4	–	–	–	0.806 (0.823)	–	–	–	0.702 (0.682)	0.597 (0.647)	0.343 (0.372)	–	0.597 (0.623)
F7	–	–	–	0.436 (0.471)	–	–	–	0.206 (0.234)	0.317 (0.344)	0.271 (0.302)	–	0.472 (0.492)
F8	–	–	–	**0.325 (0.345)**	–	–	–	0.227 (0.253)	0.298 (0.322)	**0.212 (0.263)**	–	0.466 (0.507)
C3	–	–	–	–	–	–	–	–	–	–	0.277 (0.334)	–
Cz	–	–	–	–	–	–	–	–	–	–	0.302 (0.361)	–
C4	–	–	–	–	–	–	–	–	–	–	**0.204 (0.243)**	–
T7(T3)	–	–	–	–	–	–	–	–	–	–	–	–
T8(T4)	–	–	–	–	–	–	–	–	–	–	–	–
P7(T5)	–	–	–	–	–	–	–	–	–	–	–	–
P8(T6)	–	–	–	–	–	–	–	–	–	–	–	–
P3	–	–	–	–	0.302 (0.342)	–	–	–	–	0.882 (0.963)	–	–
Pz	–	–	–	–	0.326 (0.357)	–	–	–	–	0.995 (0.917)	–	–
P4	–	–	–	–	**0.217 (0.312)**	–	–	–	–	–	–	–
O1	–	–	–	–	–	–	–	–	–	–	–	0.844 (0.924)
O2	–	–	–	–	–	–	–	–	–	–	–	0.917 (0.872)

*Correlation coefficients and mean error are not shown when smaller than 0.24 (p > 0.05) and bigger than 1.00, respectively. Each EEG spectral index, which showed the highest correlation among the brain regions, was presented the words in bold type. D, Delta; T, Theta; A, Alpha; B, Beta; G, Gamma; SA, Slow Alpha; FA, Fast Alpha; LB, Low Beta; MB, Mid Beta; HB, High Beta*.

**Figure 7:**
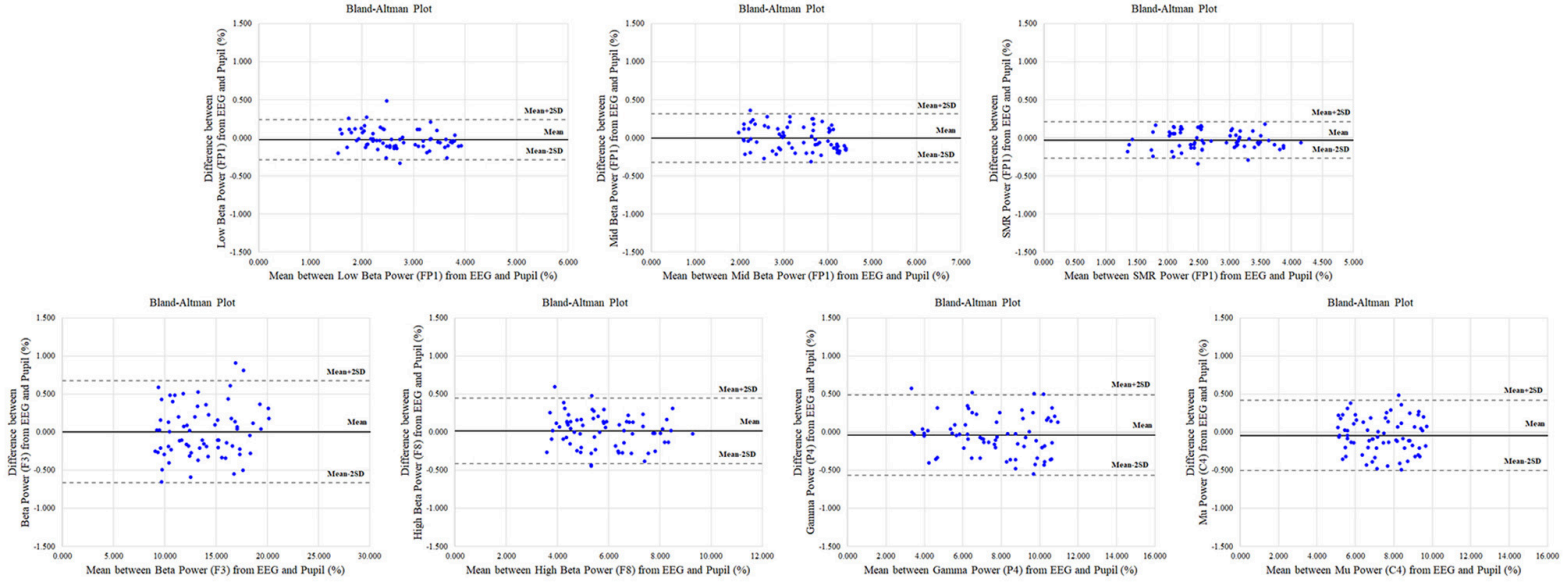
Representative Bland-Altman plots in reference condition for electroencephalogram (EEG) spectral indexes (low beta in FP1; mid beta in FP1 region; SMR in FP1; beta in F3; high beta in F8; gamma in P4; mu in C4) between pupil and EEG signals. The centre solid line represents the mean difference between two measures, with the upper and lower dotted lines representing the 95% limits (±2 SD) of agreement (*n* = 70).

**Figure 8:**
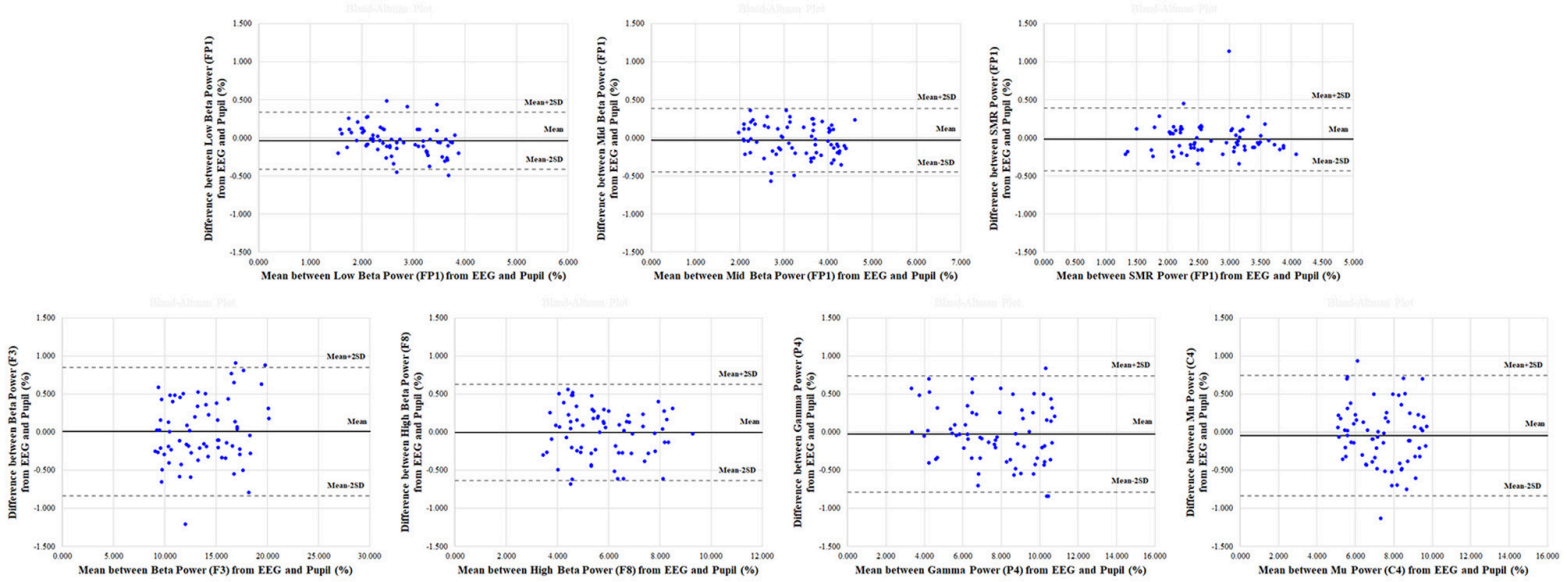
Representative Bland-Altman plots in physiological variation condition for electroencephalogram (EEG) spectral indexes (low beta in FP1; mid beta in FP1 region; SMR in FP1; beta in F3; high beta in F8; gamma in P4; mu in C4) between pupil and EEG signals. The center solid line represents the mean difference between two measures, with the upper and lower dotted lines representing the 95% limits (±2 SD) of agreement (*n* = 70).

In addition, results of the Bland-Altman analysis in reference and physiological variation conditions, shown in Figures 7, 8, confirmed that the measured values for the EEG spectral index were within the 95% limit of agreement (± 2 SD). The Bland-Altman plots demonstrated good agreement between EEG spectral indexes from the pupil and EEG signals in both conditions.

**Figure 9:**
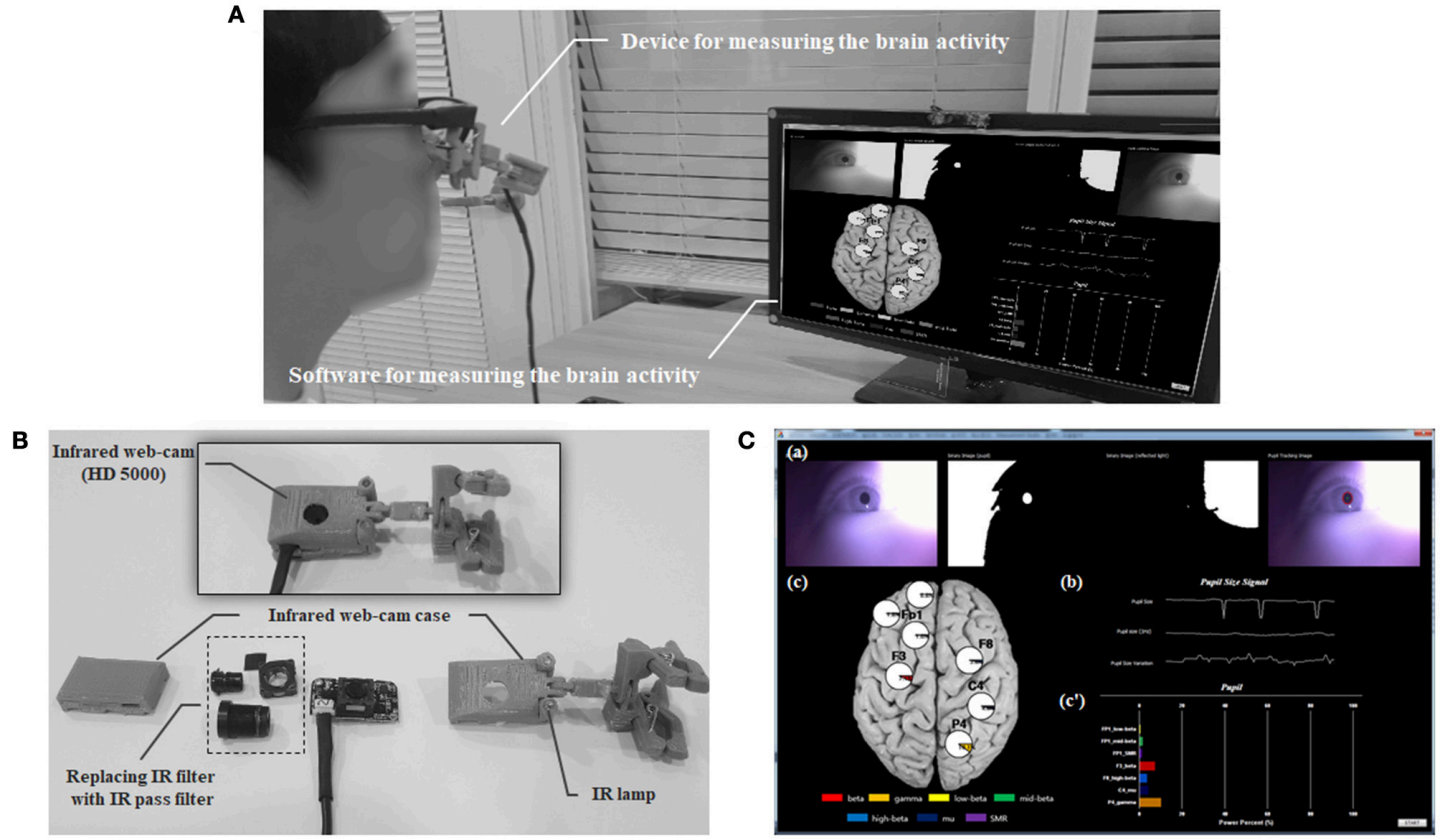
The real-time system for measuring brain activity using an infrared (IR) web-cam. **(A)** Overview the real-time system. **(B)** Configuration of the measuring device including the web-cam. **(C)** Introduction to the measuring software: (a) Protocol for detecting the pupil area; (b) Pupillary response including raw, re-sampled, and filtered pupil diameter data; (c and c′) Output of the relative power for the electroencephalogram spectral index (low beta power in the FP1 region; mid beta power in the FP1 region; SMR power in the FP1 region; beta power in the F3 region; high beta power in the F8 region; gamma power in the P4 region; mu power in the C4 region).

A real-time system for noncontact measurement the EEG spectral index was developed with the IR web-cam (Figure 9). This system comprised an IR webcam, a near-IR illuminator (IR lamp), and a personal computer for analysis. The IR web-cam was developed by reconfiguring a high-definition web-cam (HD-5000, Microsoft Inc., USA) by replacing the IR filter with an IR pass filter (Kodac Inc., USA) inside the webcam. A real-time system can be non-contact measure the relative power for low beta in FP1, mid beta in FP1 region, SMR in FP1, beta in F3, high beta in F8, gamma in P4, and mu in C4 using the IR web-cam. This system was developed using Visual C++ 2010 and OpenCV 2.4.3, and the signal processing used LabVIEW 2010 (National Instruments Inc., Austin, TX, USA).

## Discussion

The aim of the present study was to develop a noncontact system to measure the relative power of the spectral index based on pupillary rhythms. Pupillary rhythm is closely connected to brain activities based on neural pathways; this study founded that the pupillary rhythms was strongly correlated with the seven parameters of brain activity using the synchronization phenomenon in harmonic frequency (1/100 f) during varying physiological states. The correlation coefficient was very high (>0.951 [0.951–0.991]), and the mean error was very low (< 0.345% [0.148–0.345%]) in both the EEG spectral index. Regarding concurrent validity, examination of Bland-Altman plots revealed small differences in EEG spectral index between the two measurements.

Many previous studies have been reported that pupil size variation (PSV), also known as task-evoked pupillary response (TEPR), is correlated with functional brain processing such as attention, cognitive load, and memory. Previous researches demonstrated that changes in pupil diameter correlated with cortical activity in dorsal attention network (DAN) (i.e., activity in the superior colliculus and the right thalamus) (Hafed et al., 2013; Alnæs et al., 2014; Wang and Munoz, 2015; Joshi et al., 2016), locus coeruleus–norepinephrine (LC–NE) system (Gabay et al., 2011; Geva et al., 2013; Hong et al., 2014; Murphy et al., 2014; Joshi et al., 2016), and cingulate cortex (Ebitz and Platt, 2015; Joshi et al., 2016) related to attention and cognitive function. These connectivity between changes in pupil diameter and neural network from the cortical area to brainstem is interpreted by a top-down control executive network related to attention. Changes in pupil diameter results from cortical modulation between the LC-NE system in the brainstem and the neo-cortex involving the medial–ventral prefrontal cortex (MVPC), anterior cingulate cortex (ACC), and lateral prefrontal cortex (LPC) (Bush et al., 2000; MacDonald et al., 2000). The pupil diameter response has been shown with a long delay after LC-NE system is activated by the stimulus. The delay is to modulate the autonomic arousal via a top-down pathway among brainstem and cortical areas (i.e., MVPC, ACC, and LPC), and this process leads to the change in pupil size (Christ et al., 2008; Laeng et al., 2011; Niendam et al., 2012; Buckner, 2013; Geva et al., 2013; van Steenbergen and Band, 2013). Also, some study reported that changes in pupil includes a three attention networks (i.e., top-down control) related to an early component (Pa) and a prominent late component (Pe): (1) Alerting by Pa, (2) Orienting by acceleration of Pa, and (3) Executive control by Pe (Fan et al., 2002; Isaacowitz et al., 2009; Geva et al., 2013). The Pa response in pupil is associated with the recruitment of autonomic resources through alerting and attention shifting by activation of the posterior attention system. Pe's delayed activation is related to recruiting the mental resources required for executive monitoring from MVPC, ACC, and LPC (Lorist et al., 2000; Kennerley and Walton, 2011; Geva et al., 2013), and reflects the activation in LC-NE system by ACC top-down regulation (Botvinick, 2007).

In our study, the pupillary rhythms is correlated with the brain oscillations related to low beta, mid beta, high beta, mu, beta, SMR, and gamma bands, and these parameters also are known as the indicators for attention and cognitive function supported by many previous studies. For example, the SMR, mu, low beta, mid beta, and high beta waves are correlated with a high level of attention (Tansey, 1984; Egner and Gruzelier, 2001, 2004; Lee and Han, 2011; Gruzelier, 2014; Ziółkowski et al., 2014; Kim et al., 2015), and gamma waves are connected to selective attention (Brovelli et al., 2005; Jensen et al., 2007) and memory function (Pesaran et al., 2002; Howard et al., 2003; Jensen et al., 2007). The mu and beta rhythms are continuous with cognitive load or mental workload (Pfurtscheller and Klimesch, 1992; Murata, 2005; Krause et al., 2007; Tanaka et al., 2014). Above mentioned, previous studies reported that the PSV is closely connected with the neural network and the brain activity related to attention and cognition. Pupillary rhythm is involved in brain rhythm as a result of brain processing, and our findings is supported by these connectivity and previous results.

In addition, other previous studies also have been reported the directly relationship between the pupillary rhythms and the brain oscillation. Keegan and Merritt (1995) reported a significant positive correlation among beta band power and pupil diameter, and there is a negative correlation between pupil diameter and delta, theta, and alpha band powers. Other study showed a negative correlation between slow brain activities such (theta and alpha band powers) (Wang, 2013). Hong et al. (2014) showed that the pupil diameter is significant correlated with the EEG alpha rhythms based on tight coupling between attentional state and evoked neural activity. In another study reported that the alpha frequency band power in EEG is negatively correlated with the pupil dilation (Scharinger et al., 2015). A study of Vinck et al. (2015) revealed that gamma band power is strongly correlated (i.e., positive relationship) with pupil dynamics. Our results were consistent to the previous findings, showing a strong correlation between the pupillary rhythm and frequency band powers (mu, low beta, mid beta, high beta, and gamma) of similar EEG band ranges that were mentioned in the studies above. Parameters that had no correlation (delta and theta) in our current study may find a significant relationship between pupil rhythm and these band oscillation in other harmonic frequency (i.e., 1/10 f, 1/20 f, etc.), and research on this phenomena is needed through further study.

In addition, the effect of the variation of the human physiological states on the pupil response was considered in this study. A strong correlation between the pupil and the EEG oscillations was also found in the condition of the physiological change rather the neutral state. Other non-contact measurement studies reported a correlation between behavior factor and physiological response only in neutral or reference states (Loewenfeld and Lowenstein, 1993; Steinhauer et al., 2000; Verney et al., 2001; Siegle et al., 2004; Poh et al., 2011; Balakrishnan et al., 2013; Holton et al., 2013; Janssen et al., 2015). However, because non-contact measurement technology needs to be verified under physiological change in order to apply the various environments or industries. Many previous studies reported that autonomic nervous system (ANS) has been found to be closely connected with the pupillary rhythms, exhibiting repeated contraction and expansion via the sphincter and dilator muscles (Bonvallet and Zbrozyna, 1963; Loewenfeld and Lowenstein, 1993; Steinhauer et al., 2000; Verney et al., 2001; Siegle et al., 2004), and the emotional stimuli presented in this study such as arousal, relaxation, positive, and negative are closely related to the ANS (Yang et al., 2007; Levenson, 2014; Dudas et al., 2017). Also, changes in pupillary rhythms have been related to neural activity in the dorsolateral prefrontal cortex (DLPFC) (Siegle et al., 2011), and this region is known to associate with a more cognitive effort to regulate emotions for affective event (Ochsner et al., 2004; Vanderhasselt et al., 2014; Dudas et al., 2017). Our study found a strong correlation between brain and pupillary rhythms not only in the neutral state but also in the condition of physiological changes. According to previous studies, it can be interpreted by the connectivity among changes in pupillary rhythms, autonomic balance in ANS, and activation in DLPFC. Relationship between changes in pupil diameter and in emotional states (i.e., negative, positive, arousal, and neutral) has been reported by many previous researches (Pesaran et al., 2002; Partala and Surakka, 2003; Bradley et al., 2008; Geangu et al., 2011; Henderson et al., 2014; Vanderhasselt et al., 2014).

To wear the EEG electrode cap has required to the burden and the time constraints for attaching the sensor to user, and expensive sensor, and it can be restrict application in industrial fields. Our findings can be apply to the various fields related to non-contact evaluation and monitoring of the attention and the cognitive function using an inexpensive infrared web-cam without the burden for sensor attachment. For examples, our results may be applied to industrial domain such as education, neuro-feedback, ergonomics, brain computer interface (BCI), and emotional engineering as follows: (1) Evaluating immersion or concentration level of users in off- and on-line education domains. (2) Neuro-feedback training system to enhance the attention level. (3) Human assistance system to improve the usability based on monitoring for physical and mental fatigue, and drowsiness states. (4) Interface technology to control the external device using event-related (de)synchronization (ERD/ERS) and steady-state visual evoked potential (SSVEP). (5) Measuring the emotional states such as arousal, relaxation, positive, and negative. If the following limitations are complemented, there is a possibility of applying to the various fields besides the above-mentioned examples.

This study, however, had several limitations. Pupillary response is strongly influenced by blinking and ambient light. Because the pupil area is not detected during blinking, the method proposed in this study cannot directly measure brain activity; consequently, we applied a 1 Hz re-sampling protocol. If the blinking period is < 1 s, the proposed method can obtain the mean pupil diameter by calculating the mean pupil diameter during the period when the eye is open. However, if the duration of blinking is >1 s, brain activity during that period cannot be measured. To resolve this problem completely, an algorithm for restoring pupil data from an unmeasured period is required, and the protocol for measuring brain activity using reconstructed pupil data will need verification. Additionally, pupil size is very sensitive to changes in illumination. Pupil size is controlled by the occulomotor nerve innervated via the sphincter (i.e., parasympathetic nerve) and the ophthalmic nerve is innervated via dilator muscles (i.e., sympathetic nerve) according to changes in illumination (Bonvallet and Zbrozyna, 1963; Usui and Shirakashi, 1982; Loewenfeld and Lowenstein, 1993; Steinhauer et al., 2000; Verney et al., 2001; Siegle et al., 2004; Kozicz et al., 2011; Andreassi, 2013; Júnior et al., 2015). Because measurement of brain activity was based on pupillary rhythm (i.e., pupil size variation) in this study, the proposed method may be vulnerable to errors in rapid light alterations. In a previous study, the effect of rapid light changes (550, 350, 150, 40, and 2 lux) on pupil size was tested, and respective pupil sizes were 3.5, 4.2, 5.2, 5.03, and 5.4 mm. Pupil size has not been shown to be significantly affected by illumination conditions < 150 lux (Maqsood, 2017). The ambient light in this experiment was measured to be in the in range of 135–160 lux (mean 147.42 ± 6.02 lux) using a commercially available device (Visible Light SD Card Logger, Sper Scientific Meters Ltd., Scottsdale, AZ, USA), which did not affect the measurement of brain activity using the proposed method. However, in experimental design of this study, condition for the ambient light was not considered with presenting the sound instead of visual stimuli. Thus, proposed method in this study cannot be applied to the condition what the ambient light changes rapidly. In order to resolve this issue, further research investigating the relationship between changes in pupil diameter and a rapid change in ambient light intensity is required before rigorous application in various fields.

In this study brain activity was measured using a low-cost IR webcam. Pupillary rhythms with harmonic frequency (1/100 f) revealed a high correlation in our protocol with seven parameters (relative powers) of the EEG spectral index [i.e., low beta (FP1), mid beta (FP1), SMR (FP1), beta (F3), high beta (F8), gamma (P4), and mu (C4)] during the changes of the physiological states (arousal, relaxation, positive, negative, and neutral states). However, in order for the proposed method to be applied in various fields, the method needs to be investigated under different conditions (rapid illumination changes, other physiologic conditions, or other stimuli for causing physiological state) that were not included in the current protocol. To overcome the above limitations in further studies, the proposed method may measure and evaluate brain activity in various application fields using a simple, low-cost, non-contact system. Because measurement of brain activity using sensors has traditionally required complex and expensive equipment, and involved the inconveniences and burden of sensor attachment.

## Author contributions

SP and MW designed the study with investigation of previous studies and performed the experiments. SP performed the data analysis and verification for statistical significance, and wrote the manuscript with support from MW. SP and MW discussed the results and contributed to the final manuscript. MW conceived the study and was in charge of overall direction and planning.

### Conflict of interest statement

The authors declare that the research was conducted in the absence of any commercial or financial relationships that could be construed as a potential conflict of interest.
